# Velamentous cord insertion in singleton pregnancies: ultrasound diagnostic accuracy, risk factor analysis, and perinatal outcomes

**DOI:** 10.3389/fmed.2026.1802669

**Published:** 2026-04-01

**Authors:** Nan Liang, Hongzhi Du, Jialei Xu, Liling Shi

**Affiliations:** Department of Ultrasound, Shanxi Children’s Hospital (Shanxi Women and Children Hospital), Taiyuan, Shanxi, China

**Keywords:** anemia, assisted reproductive technology, color Doppler ultrasound, pregnancy outcomes, singleton pregnancy, vasa previa, velamentous cord insertion

## Abstract

**Background:**

Velamentous cord insertion (VCI) complicates 0.9–1.9% of singleton pregnancies and is associated with adverse perinatal outcomes due to unprotected fetal vessels traversing the membranes. Color Doppler ultrasound enables prenatal diagnosis, yet systematic evaluation of diagnostic performance and risk factor association models remains limited. We hypothesized that systematic second-trimester screening achieves superior diagnostic accuracy before 28 weeks, and that identification of clinical risk factors associated with VCI would enhance understanding of its etiology and inform screening strategies.

**Methods:**

This retrospective case–control study included 240 singleton pregnancies (120 confirmed VCI cases and 120 age-matched controls) between 2022–2025. All underwent systematic second-trimester color Doppler ultrasound evaluating cord insertion site, membranous vessel length, and vasa previa. Multivariate logistic regression was used to identify independent risk factors associated with VCI and to develop a risk factor association model; model discrimination was assessed using C-index with 1,000-bootstrap internal validation.

**Results:**

Color Doppler ultrasound achieved 94.2% sensitivity and 100% specificity for VCI detection, with superior accuracy before 28 weeks (97.3% vs. 85.7%, *p* = 0.008). Three independent risk factors were identified: Three independent clinical risk factors were identified: ART (aOR = 2.87), maternal anemia (aOR = 2.15), and short cervical length (aOR = 2.34). Placenta previa demonstrated a strong association with VCI (OR = 3.92), likely reflecting a shared spectrum of abnormal trophoblast implantation. The multivariate model demonstrated moderate discrimination (C-index = 0.713, 95% CI: 0.648–0.778) for distinguishing VCI from controls. The VCI cohort was associated with significantly higher rates of adverse outcomes: preterm delivery 22.5% vs. 8.3% (*p* = 0.002), FGR 18.3% vs. 6.7% (*p* = 0.004), and perinatal mortality 3.3% vs. 0% (*p* = 0.042).

**Conclusion:**

In tertiary referral settings with experienced operators, targeted color Doppler ultrasound demonstrates high diagnostic accuracy for VCI detection, particularly during second-trimester evaluation. The developed risk factor model demonstrated moderate discrimination in this cohort; external validation in independent populations is required before clinical implementation.

## 1 Introduction

Velamentous cord insertion (VCI), defined as umbilical vessel insertion into the fetal membranes distant from the placental margin, complicates 0.9–1.9% of singleton pregnancies (1). Unlike the typical central insertion, the exposed vessels—deprived of protective Wharton’s jelly—traverse the amniochorionic membranes unprotected, rendering them vulnerable to compression, rupture, and thrombosis during labor or membrane rupture (2). These mechanical vulnerabilities predispose to catastrophic complications including vasa previa, fetal hemorrhage, and intrauterine demise, often with minimal premonitory symptoms (3, 4).

Beyond immediate mechanical risks, aberrant vascular architecture in VCI impairs fetoplacental hemodynamics, potentially contributing to uteroplacental insufficiency and subsequent fetal growth restriction (FGR) (1). Prenatal diagnosis relies fundamentally on systematic ultrasound visualization of the cord insertion site, with color Doppler imaging serving as the cornerstone for identifying the characteristic fan-like vessel arborization (5). However, diagnostic accuracy is highly dependent on gestational age, operator experience, and technical parameters, with the optimal screening window remaining debated (6, 7). Consequently, VCI remains under-recognized in routine obstetric practice, partly due to inconsistent screening protocols and variable diagnostic proficiency among sonographers (8).

While epidemiological studies have linked VCI to assisted reproductive technology (ART), maternal anemia, and placental malformations, these associations derive predominantly from retrospective cohorts lacking adequate controls or multivariate adjustment (9, 10). Consequently, the independent contribution of each risk factor remains undefined, and—critically—no validated risk factor association model exists for singleton pregnancies, limiting identification of high-risk subpopulations requiring enhanced surveillance (11).

Despite the established association between VCI and adverse perinatal outcomes, substantial uncertainties persist regarding the diagnostic accuracy of color Doppler ultrasound across gestational ages and the lack of validated tools for individualized risk factor analysis (1, 2). Prior studies have been limited by small sample sizes, inadequate control for confounders such as placental location and conception mode, and insufficient characterization of the differential impact of coexistent vasa previa on perinatal morbidity (4, 6). Consequently, evidence-based management remains hindered by the absence of robust predictive models integrating sonographic and maternal parameters.

To address these limitations, this retrospective case–control study aimed to systematically quantify color Doppler diagnostic performance by gestational age, and to identify independent maternal and obstetric risk factors associated with VCI through rigorous multivariate adjustment. We further sought to evaluate the specific risk amplification associated with coexistent vasa previa to inform delivery planning. We hypothesized that systematic second-trimester screening achieves superior diagnostic accuracy and that identification of clinical risk factors associated with VCI would enhance understanding of its etiology and inform screening strategies.

## 2 Methods

### 2.1 Study design

This retrospective case–control study was conducted at the Department of Obstetrics and Gynecology between January 2022 and December 2025. The institutional review board approved the study protocol (No: IRB-KYYN-2023-011), which complied with the ethical principles of the Declaration of Helsinki. Given the retrospective nature of the investigation, the requirement for written informed consent was waived. All patient data were de-identified prior to analysis to ensure confidentiality and privacy protection.

### 2.2 Study population

The study cohort comprised two groups of singleton pregnant women. The case group included 120 patients with prenatal ultrasound suspicion of velamentous cord insertion that was subsequently confirmed by postpartum pathological examination of the placenta. The control group consisted of 120 women with centrally inserted umbilical cords, randomly selected using computer-generated random numbers (Excel RAND function) from the delivery database and frequency-matched to cases by maternal age (±2 years) during the identical study period. All participants were required to have complete medical records and comprehensive obstetric ultrasound imaging data available for review. Women were excluded if they had multiple pregnancies, known fetal chromosomal abnormalities, major congenital malformations detected before VCI diagnosis, severe maternal comorbidities including cardiovascular disease, renal insufficiency, or active infectious diseases, or if they were lost to follow-up before delivery. Sample size calculation was performed based on the primary outcome of preterm delivery (<37 weeks), expecting 22.5% in the VCI group versus 8.3% in controls based on pilot data (absolute difference = 14.2%). Using an alpha level of 0.05 and statistical power of 80% (two-sided), the minimum required sample size was determined to be 110 subjects per group, with our enrollment of 120 per group providing additional statistical robustness.

### 2.3 Ultrasound protocol

All ultrasound examinations were performed by experienced obstetric sonographers using either a GE Voluson E10 or Philips E10 system equipped with 2–9 MHz convex transducers. Sonographers were not blinded to maternal clinical information during routine clinical examinations, as imaging was performed as part of standard clinical care. However, for the subset of 30 cases used for inter-observer agreement assessment, two independent sonographers were blinded to each other’s assessments and to the final diagnosis. Given the retrospective nature of this study and the use of clinical imaging data, complete blinding to all maternal risk factors was not feasible; we acknowledge this as a potential source of detection bias. To mitigate this, all ultrasound measurements were performed using standardized protocols prior to knowledge of final VCI status, and the primary diagnostic criterion (cord insertion site >2 cm from placental margin with fan-like vessel distribution) is an objective anatomic finding less susceptible to observer bias.

**Figure 1:**
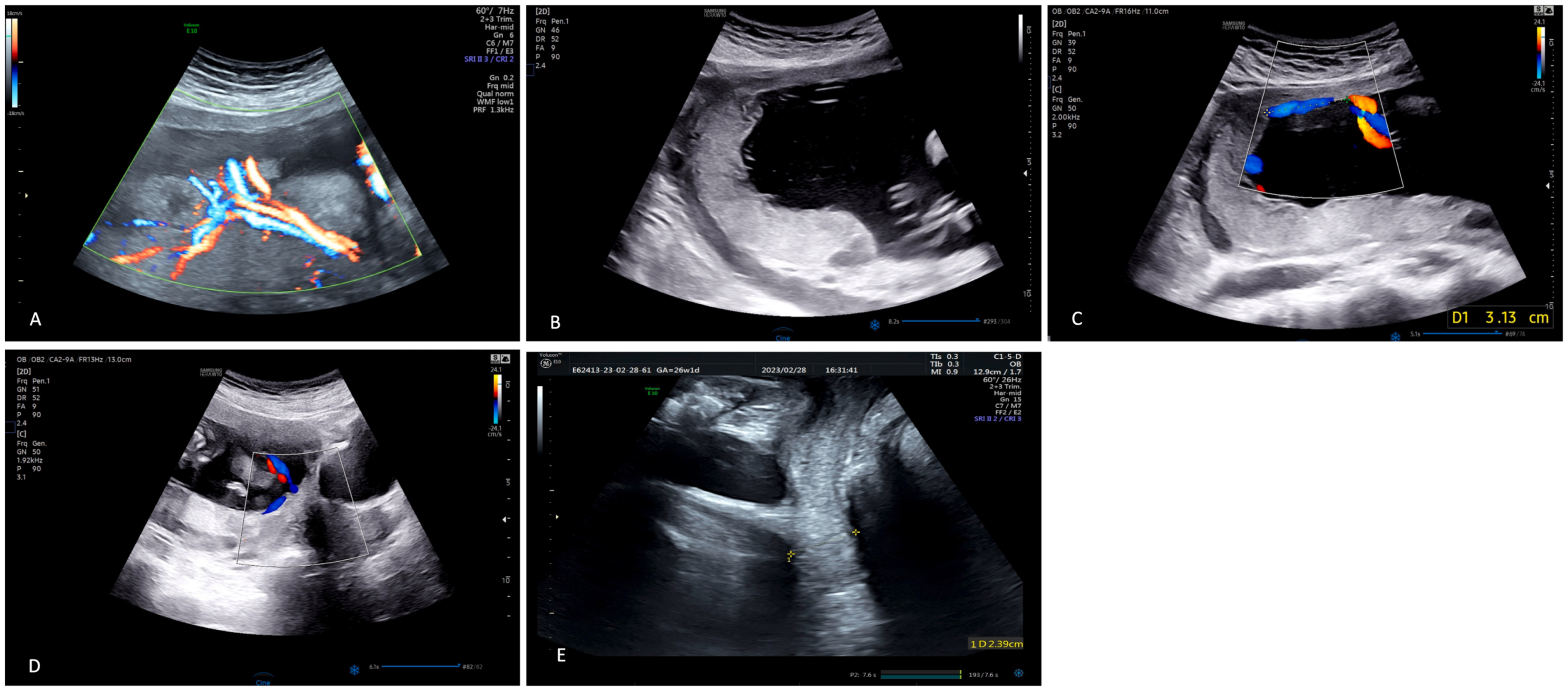
Representative ultrasound images of VCI diagnosis. **(A)** Color Doppler (GE Voluson E10, 23 + 0 weeks) showing fan-like vessel distribution. **(B)** Gray-scale (Samsung HERA W10, 22 + 2 weeks) demonstrating cord insertion >2 cm from placental margin. **(C)** Measurement of intramembranous length (31.3 mm). **(D)** Vasa previa detection (17 + 3 weeks). **(E)** Cervical length measurement (23.9 mm).

Comprehensive placental assessment was conducted during the second trimester between 18 and 28 weeks gestation, as this window represents the optimal period for reliable visualization of cord insertion anatomy before fetal crowding and decreased amnionic fluid may obscure details. In cases with suspected VCI, follow-up examinations were performed at 32–34 weeks to confirm persistence of the abnormal insertion and to screen for complications such as vasa previa. The ultrasound protocol began with grayscale imaging to systematically map the chorionic plate and identify the precise umbilical cord insertion site. Scanning planes were oriented perpendicular to the placental margin to detect any cord insertion located greater than 2 cm from the placental edge. Color Doppler imaging was then activated using low velocity scale settings (10–20 cm/s), high color gain, and minimal wall motion filter to maximize sensitivity for slow-flow vessels. The sonographer traced the umbilical vessels from their insertion point to the chorionic plate, documenting the characteristic fan-like arborization pattern through the free membranes and confirming the absence of protective Wharton’s jelly. Spectral Doppler analysis was employed to sample flow within intramembranous vessels, recording umbilical artery resistance index (RI), pulsatility index (PI), and systolic/diastolic ratio. When vessels were detected traversing the internal cervical os, transvaginal or transperineal ultrasound with color Doppler was performed to evaluate for vasa previa. A definitive prenatal diagnosis of VCI required fulfillment of two criteria: color Doppler visualization of umbilical cord insertion into the free fetal membranes more than 2 cm from the placental margin with vessels splaying in a fan-like distribution without Wharton’s jelly protection, and postpartum macroscopic examination revealing cord insertion at the membrane-chorionic junction with histopathological verification of exposed vessels within the amniochorionic membranes. To ensure reproducibility of VCI diagnosis, 30 random cases were independently reviewed by two blinded sonographers (each with >5 years of obstetric ultrasound experience). Inter-observer agreement was assessed using Cohen’s Kappa coefficient (*κ* = 0.85, 95% CI: 0.72–0.98), indicating excellent agreement beyond chance. In addition to placental assessment, transvaginal ultrasound was performed to measure cervical length using standardized techniques. Measurements were obtained with the cervix visualized in the sagittal plane, with the calipers placed at the internal and external os. Three measurements were recorded and the shortest value was used for analysis. Cervical length <25 mm was defined as short cervix. Representative images are shown in Figure 1.

### 2.4 Data collection

Clinical and demographic variables were extracted from electronic medical records and included maternal age, body mass index (BMI) at booking, parity, and conception method categorized as spontaneous or ART. Obstetric history encompassed previous miscarriages, uterine anomalies, and prior placental disorders. Pregnancy complications were recorded according to standardized diagnostic criteria, including anemia (defined as hemoglobin concentration <100 g/L), gestational diabetes mellitus, and hypertensive disorders of pregnancy. Placental characteristics such as placenta previa and succenturiate lobes were documented. Quantitative ultrasound measurements included the distance between the cord insertion site and the nearest placental edge in millimeters, the length of the intramembranous vascular segment, and the presence or absence of vasa previa. Doppler indices of umbilical artery flow were recorded as described above.

Pregnancy outcomes were tracked through delivery and the immediate postpartum period, with maternal parameters comprising mode of delivery, estimated blood loss (with postpartum hemorrhage defined as >500 mL), and requirement for manual placental removal. Neonatal outcomes included gestational age at delivery, birth weight, Apgar scores at 1 and 5 min, admission to the neonatal intensive care unit (NICU), fetal growth restriction (FGR, defined as birth weight <10th percentile for gestational age), congenital anomalies detected after birth, and perinatal mortality.

### 2.5 Statistical analysis

All statistical analyses were conducted using R software version 4.1.2. Prior to multivariate model development, collinearity diagnostics were performed using variance inflation factors (VIF). All candidate variables demonstrated acceptable collinearity (VIF < 2.5), well below the conventional threshold of 5.0, with the highest VIF observed for maternal age (VIF = 1.8) and ART (VIF = 1.6). No variables required exclusion based on collinearity. Sensitivity analyses were performed using multiple imputation by chained equations (MICE, m = 5) for covariates with missing values, assuming data were missing at random. The proportion of missing data was assessed for all study variables. Overall, missing data were minimal (<5% for all variables), occurring in cervical length measurements (1.7%), maternal BMI (1.3%), and prior miscarriage history (0.8%). No missing data were observed for key outcomes (VCI status, delivery gestational age, birth weight) or core exposures (ART, anemia, placenta previa). Given the low proportion of missing data, complete-case analysis was used as the primary approach, with multiple imputation performed as a sensitivity analysis to confirm robustness of findings. Imputation models included all variables in the multivariate analysis and outcome variables. Pooled estimates from 5 imputed datasets were compared with complete-case analysis. Continuous variables were assessed for normality using the Shapiro–Wilk test and presented as either mean ± standard deviation or median with interquartile range, as appropriate. Categorical variables were expressed as frequencies and percentages. Between-group comparisons were performed using independent *t*-tests or Mann–Whitney U tests for continuous data and chi-square or Fisher’s exact tests for categorical variables, with statistical significance defined as a two-tailed *p*-value <0.05. Risk factor analysis proceeded in two stages. First, univariate analysis was performed to screen each variable for association with VCI. Variables demonstrating *p* < 0.10 in this initial screening, along with clinically established confounders (maternal age >35 years, nulliparity, and prior miscarriage) that were forced into the model regardless of univariate significance, were entered into a multivariate logistic regression model. With 120 VCI cases (events) and 8 candidate variables, the events-per-variable ratio was 15:1, exceeding the minimum threshold of 10:1 recommended for reliable logistic regression estimates. Backward stepwise selection was employed with entry and exit criteria of *p* < 0.10 and *p* > 0.05, respectively, to identify independent risk factors associated with VCI while adjusting for potential confounders. Given the case–control design, this analysis identifies risk factors associated with VCI rather than predicting absolute risk. The C-index reflects discrimination between cases and controls, not prediction in a general obstetric population. Model discrimination was assessed using the concordance index (C-index), with internal validation performed through 1,000 bootstrap resamples to correct for optimism. Results of the regression analysis were reported as odds ratios (ORs) with 95% confidence intervals (CIs), and model goodness-of-fit was evaluated using the Hosmer-Lemeshow test. The diagnostic performance of prenatal ultrasound for VCI detection was quantified by calculating sensitivity, specificity, positive predictive value, and negative predictive value. Receiver operating characteristic (ROC) curve analysis was employed to determine optimal cutoff values for quantitative ultrasound parameters.

## 3 Results

### 3.1 Study population

**Figure 2:**
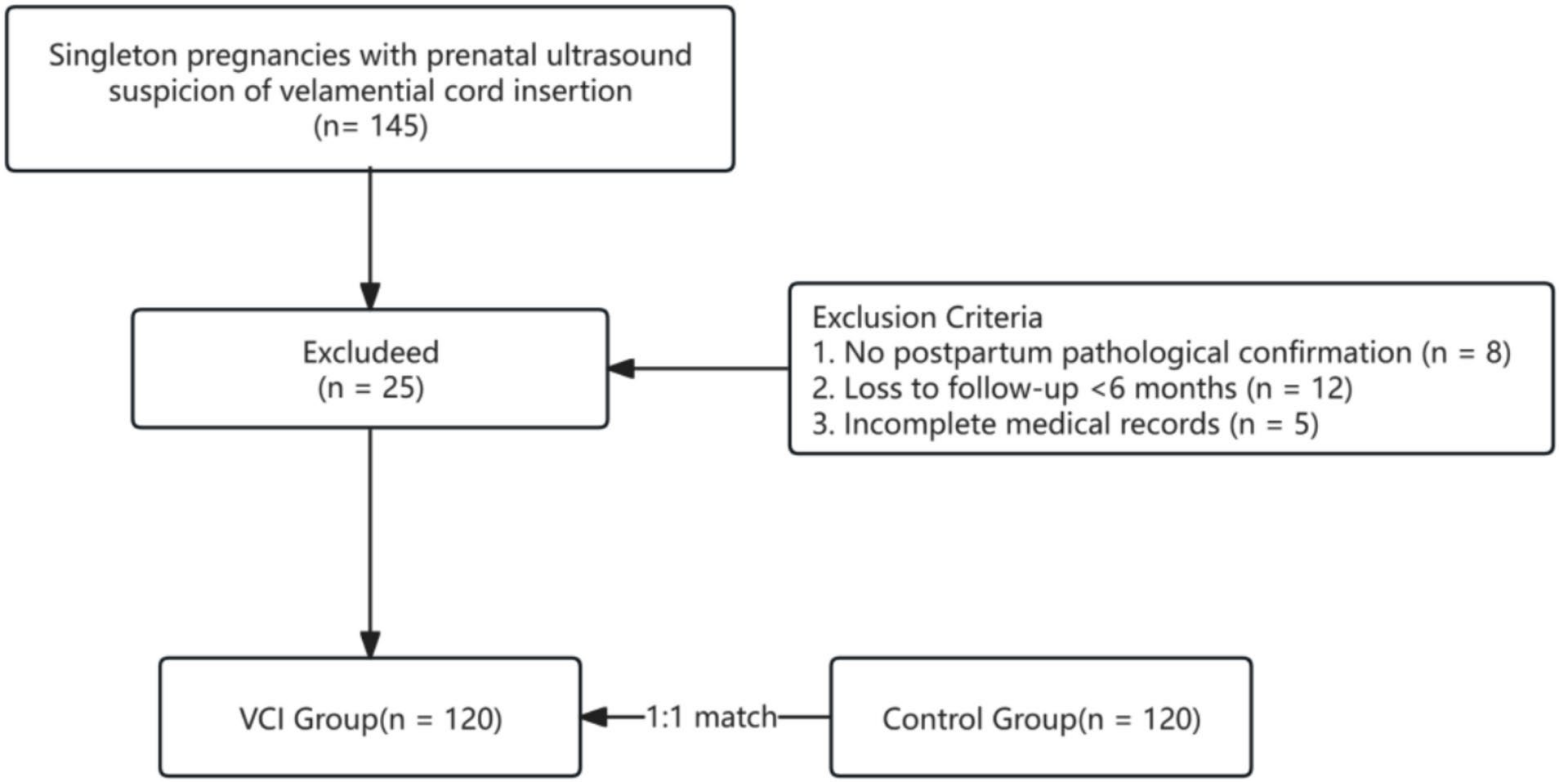
Patient enrollment and screening.

During the study period (January 2022–December 2025), 145 singleton pregnancies with prenatal ultrasound suspicion of velamential cord insertion (VCI) were identified from our institutional database. Of these, 25 were excluded: 8 had no postpartum pathological confirmation, 12 were lost to follow-up before delivery, and 5 had incomplete medical records. The remaining 120 confirmed VCI cases were included and matched 1:1 with controls (Figure 2). The VCI and control groups demonstrated comparable baseline demographics (Table 1). No significant differences were observed in maternal age (31.8 ± 4.2 vs. 31.2 ± 3.9 years, *p* = 0.432) or BMI (23.6 ± 3.8 vs. 23.1 ± 3.5 kg/m^2^, *p* = 0.287). Categorical variables revealed significant between-group differences: ART conception was more prevalent in the VCI cohort (34 [28.3%] vs. 15 [12.5%], χ^2^ = 9.63, *p* = 0.003), as was maternal anemia (42 [35.0%] vs. 22 [18.3%], χ^2^ = 8.57, *p* = 0.004). Placenta previa occurred in 18 (15.0%) VCI cases versus 5 (4.2%) controls (χ^2^ = 8.24, *p* = 0.003). Cervical length was significantly shorter in the VCI group [32.4 mm (IQR: 28.6–36.2) vs. 36.7 mm (33.1–39.8), *p* < 0.001], with short cervix (<25 mm) occurring in 15.0% vs. 4.2% (*p* = 0.003).

**Table 1:** Baseline maternal and pregnancy characteristics.

Characteristic	VCI group (*n* = 120)	Control group (*n* = 120)	*P*-value	Distribution
Maternal age (years)	31.8 ± 4.2	31.2 ± 3.9	0.432	Normal
Maternal BMI (kg/m^2^)	23.6 ± 3.8	23.1 ± 3.5	0.287	Normal
Nulliparity	54 (45.0%)	48 (40.0%)	0.418	
Assisted reproductive technology	34 (28.3%)	15 (12.5%)	0.003	
Prior miscarriage	28 (23.3%)	22 (18.3%)	0.332	
Hemoglobin <100 g/L	42 (35.0%)	22 (18.3%)	0.004	
Gestational diabetes mellitus	16 (13.3%)	11 (9.2%)	0.287	
Hypertensive disorders	9 (7.5%)	6 (5.0%)	0.406	
Placenta previa	18 (15.0%)	5 (4.2%)	0.003	
Succenturiate placental lobe	8 (6.7%)	3 (2.5%)	0.089	
Gestational age at first US (weeks)	20.3 ± 4.5	20.8 ± 4.1	0.355	Normal
Cervical length (mm)	32.4 (28.6–36.2)	36.7 (33.1–39.8)	<0.001	Non-normal
Short cervix (<25 mm)	18 (15.0%)	5 (4.2%)	0.003	

Values are mean ± standard deviation for normal distribution, median (interquartile range) for non-normal distribution, or *n* (%). Distribution was assessed using the Shapiro–Wilk test. BMI, body mass index; US, ultrasound; VCI, velamentous cord insertion.

### 3.2 Ultrasound diagnostic performance and measurements

Color Doppler ultrasound correctly identified VCI prenatally in 113 of 120 cases, yielding an overall sensitivity of 94.2% (95% CI: 88.7–97.4%), specificity of 100%, positive predictive value of 100%, and negative predictive value of 95.2%.

Diagnostic accuracy was significantly higher when examinations were performed before 28 weeks gestation (sensitivity 97.3% vs. 85.7% after 28 weeks, *p* = 0.008). Quantitative ultrasound measurements revealed that the mean distance from cord insertion to the nearest placental edge was 45.3 ± 12.1 mm in the VCI group, with an average intramembranous vascular length of 38.6 ± 10.4 mm. The characteristic fan-like vessel distribution was observed in 94.2% of VCI cases, while vasa previa was identified sonographically in 15 cases (12.5%). Umbilical artery Doppler indices were within normal ranges in both groups, with no significant differences in resistance index (RI) (0.68 ± 0.08 vs. 0.66 ± 0.07, *p* = 0.087) or pulsatility index (PI) (1.12 ± 0.21 vs. 1.08 ± 0.19, *p* = 0.123) between the VCI and control cohorts (Table 2).

**Table 2:** Ultrasonic diagnostic performance and measurements.

Parameter	Value
Diagnostic performance (*n* = 120)	
Sensitivity (95% CI)	94.2% (88.7–97.4%)
Specificity	100%
Positive Predictive Value	100%
Negative Predictive Value	95.2%
Sensitivity by gestational age	
≤28 weeks (*n* = 92)	97.3%
>28 weeks (*n* = 28)	85.7%
*P*-value for difference	0.008
Quantitative measurements (VCI Group)	
Distance to placental edge (mm)	45.3 ± 12.1
Intramembranous vascular length (mm)	38.6 ± 10.4
Qualitative features	
Fan-like vessel distribution	113 (94.2%)
Vasa previa detected	15 (12.5%)
Doppler indices (VCI vs. Control)	
Umbilical artery RI	0.68 ± 0.08 vs. 0.66 ± 0.07
Umbilical artery PI	1.12 ± 0.21 vs. 1.08 ± 0.19
*P*-value for RI difference	0.087
*P*-value for PI difference	0.123

RI, resistance index; PI, pulsatility index; VCI, velamentous cord insertion.

### 3.3 Association between VCI and pregnancy outcomes

Sensitivity analyses using multiple imputation demonstrated minimal variation in effect estimates for all predictors compared with complete-case analysis (Supplementary Table 1). The relative differences ranged from −3.3 to +3.0%, with pooled C-index of 0.709 (95% CI: 0.645–0.773) versus 0.713 in the complete-case analysis, confirming the robustness of our findings against potential bias from missing data. The VCI group delivered at significantly lower gestational age (37.6 [IQR: 35.4–39.1] vs. 39.2 [38.5–40.0] weeks, *p* < 0.001) and birth weight (2,980 [2650–3,240] vs. 3,240 [2980–3,480] g, *p* < 0.001) (Table 3). Categorical outcomes analyzed via chi-square tests revealed higher rates of preterm delivery <37 weeks (27 [22.5%] vs. 10 [8.3%], χ^2^ = 9.47, *p* = 0.002) and FGR (22 [18.3%] vs. 8 [6.7%], χ^2^ = 7.92, *p* = 0.005). Cesarean section was performed in 82 (68.3%) VCI cases versus 39 (32.5%) controls (χ^2^ = 28.74, *p* < 0.001). NICU admissions (32 [26.7%] vs. 14 [11.7%], χ^2^ = 8.73, *p* = 0.003) and respiratory distress syndrome (17 [14.2%] vs. 4 [3.3%], χ^2^ = 8.92, *p* = 0.003) were significantly increased. Perinatal mortality was significantly higher in the VCI group (3.3% vs. 0%; risk difference 3.3, 95% CI: 0.9–8.2%; *p* = 0.042). Due to zero events in controls, relative risk could not be calculated. Postpartum hemorrhage >500 mL was more frequent in the VCI group (20 [16.7%] vs. 9 [7.5%], χ^2^ = 5.21, *p* = 0.022).

**Table 3:** Comparison of maternal and neonatal outcomes.

Outcome	VCI group (*n* = 120)	Control group (*n* = 120)	*P*-value
Gestational age at delivery (weeks)	37.6 (35.4–39.1)	39.2 (38.5–40.0)	<0.001
Preterm delivery <37 weeks	27 (22.5%)	10 (8.3%)	0.002
Preterm delivery <34 weeks	12 (10.0%)	3 (2.5%)	0.009
Birth weight (g)	2,980 (2,650–3,240)	3,240 (2,980–3,480)	<0.001
FGR (<10th percentile)	22 (18.3%)	8 (6.7%)	0.004
Cesarean section	82 (68.3%)	39 (32.5%)	<0.001
NICU admission	32 (26.7%)	14 (11.7%)	0.003
Apgar score <7 at 1 min	19 (15.8%)	7 (5.8%)	0.009
Apgar score <7 at 5 min	6 (5.0%)	2 (1.7%)	0.089
Respiratory distress syndrome	17 (14.2%)	4 (3.3%)	0.001
Congenital anomalies detected	8 (6.7%)	3 (2.5%)	0.089
Perinatal death	4 (3.3%)	0 (0%)	0.042
Postpartum hemorrhage >500 mL	20 (16.7%)	9 (7.5%)	0.019
Manual placental removal	11 (9.2%)	6 (5.0%)	0.157

Data are presented as median (IQR) or *n* (%). Continuous variables were non-normally distributed (Shapiro–Wilk *P* < 0.05). FGR, fetal growth restriction; NICU, neonatal intensive care unit; VCI, velamentous cord insertion.

Multivariate logistic regression using backward stepwise selection identified three independent clinical risk factors associated with VCI (Table 4): ART conception (adjusted OR = 2.87, 95% CI: 1.48–5.56, *p* = 0.002), maternal anemia (adjusted OR = 2.15, 95% CI: 1.14–4.05, *p* = 0.018), and short cervical length (adjusted OR = 2.34, 95% CI: 1.21–4.53, *p* = 0.011). Placenta previa demonstrated a strong association with VCI (adjusted OR = 3.92, 95% CI: 1.82–8.43, *p* < 0.001); however, given the shared pathophysiology of abnormal trophoblast implantation, this likely represents a correlated placental condition rather than an independent predictor. Model discrimination for the three-factor clinical model was moderate with a C-index of 0.713 (95% CI: 0.648–0.778).

**Table 4:** Univariate and multivariate logistic regression analysis of VCI risk factors.

Risk factor	Univariate OR (95% CI)	*P*-value	Multivariate OR (95% CI)	*P*-value
Maternal age >35 years	1.72 (1.08–2.73)	0.021	1.56 (0.94–2.59)	0.086
Assisted reproductive technology	2.76 (1.44–5.31)	0.002	2.87 (1.48–5.56)	0.002
Hemoglobin <100 g/L	2.35 (1.30–4.26)	0.005	2.15 (1.14–4.05)	0.018
Placenta previa	4.01 (1.47–10.98)	0.006	3.92 (1.82–8.43)	<0.001
Short cervix (<25 mm)	2.78 (1.15–5.56)	0.005	2.34 (1.21–4.53)	0.011
Prior miscarriage	1.35 (0.73–2.49)	0.342	–	–
Nulliparity	1.22 (0.74–2.03)	0.432	–	–

Multivariate model 1 (clinical risk factors) included ART, maternal anemia, and short cervical length. Placenta previa was analyzed separately given its likely shared pathophysiology with VCI. Model 1 discrimination: C-index = 0.713 (95% CI: 0.648–0.778).

### 3.4 Efficacy and clinical utility

**Figure 3:**
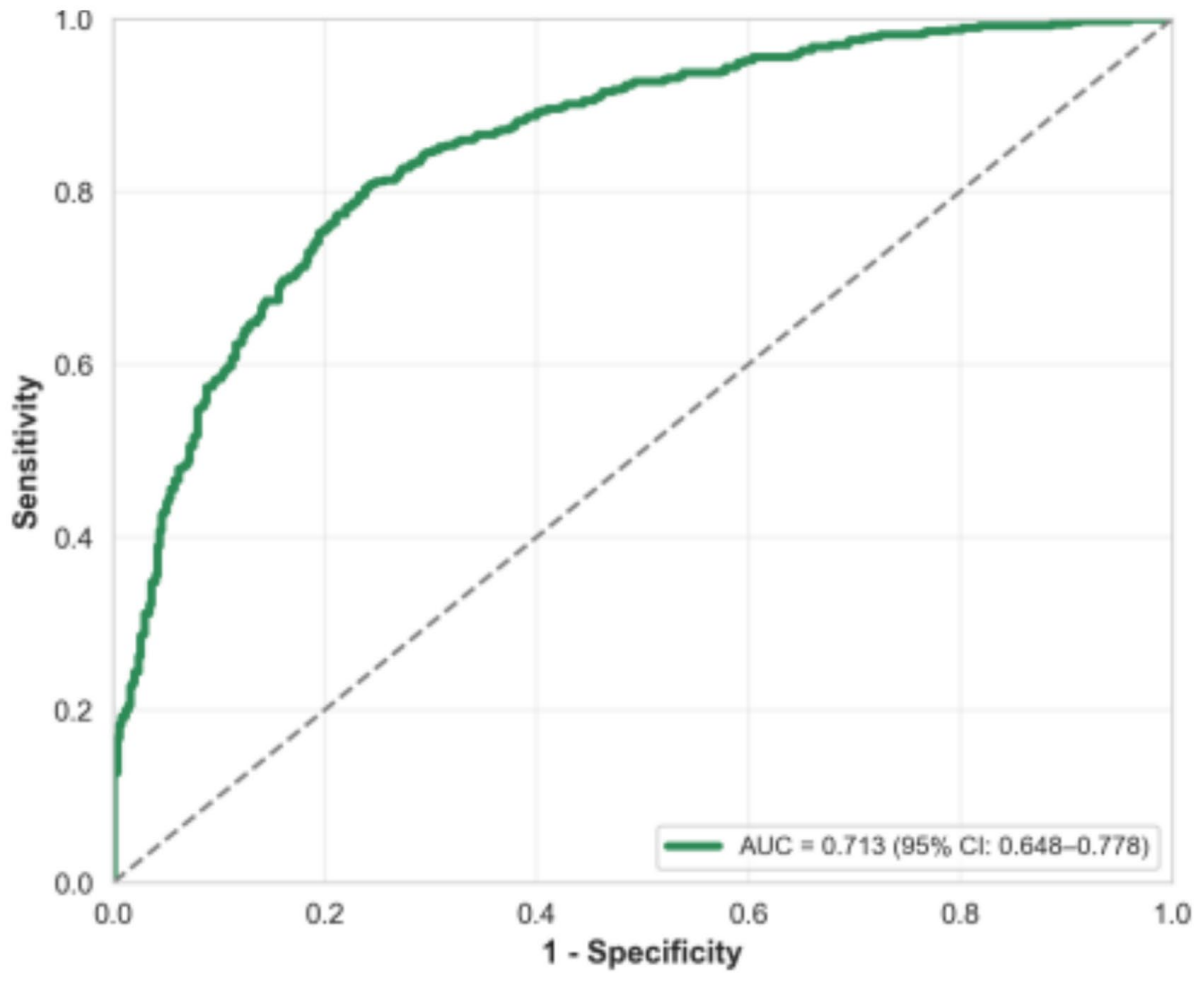
Receiver operating characteristic (ROC) curve of the multivariate model for distinguishing velamentous cord insertion cases from controls. The area under the curve (AUC) is 0.713 (95% CI: 0.648–0.778).

The three-factor clinical risk model (ART, maternal anemia, short cervical length) achieved an area under the ROC curve of 0.713 (95% CI: 0.648–0.778) for distinguishing VCI cases from controls (Figure 3). Addition of placenta previa to the model increased the C-index marginally to 0.738; however, given its status as a correlated placental condition rather than an independent clinical predictor, we present the three-factor model as the primary clinical tool.

### 3.5 Subgroup analysis by vasa previa status

Stratified analysis compared outcomes between VCI with vasa previa (*n* = 15) and isolated VCI (*n* = 105) (Table 5). Continuous variables were compared using Mann–Whitney U tests due to non-normal distribution, and categorical variables by chi-square or Fisher’s exact tests. The vasa previa subgroup delivered earlier (median 35.4 [IQR: 34.2–36.5] vs. 37.8 [36.1–39.3] weeks, *p* = 0.008) with higher rates of preterm delivery (8 [53.3%] vs. 19 [18.1%], χ^2^ = 8.43, *p* = 0.004) and perinatal mortality (2 [13.3%] vs. 2 [1.9%], Fisher’s *p* = 0.026). All vasa previa cases required cesarean delivery (15 [100%] vs. 67 [63.8%], χ^2^ = 6.73, *p* = 0.010). NICU admission rates were elevated (7 [46.7%] vs. 25 [23.8%], χ^2^ = 3.89, *p* = 0.049). FGR incidence did not differ between subgroups (20.0% vs. 18.1%, *p* = 0.846).

**Table 5:** Pregnancy outcomes in VCI cases vasa previa status.

Outcome	VCI + Vasa previa (*n* = 15)	VCI alone (*n* = 105)	*P*-value
Gestational age at delivery (weeks)	35.2 ± 2.1	37.5 ± 2.6	0.008
Preterm delivery <37 weeks	8 (53.3%)	19 (18.1%)	0.003
Cesarean section	15 (100%)	67 (63.8%)	0.004
Vaginal delivery attempted	0 (0%)	65 (61.9%)	<0.001
Birth weight (g)	2,650 ± 580	3,020 ± 490	0.012
Fetal growth restriction	3 (20.0%)	19 (18.1%)	0.846
Perinatal death	2 (13.3%)	2 (1.9%)	0.026
NICU admission	7 (46.7%)	25 (23.8%)	0.049
Respiratory distress syndrome	4 (26.7%)	13 (12.4%)	0.128

Due to the limited sample size in the vasa previa subgroup (*n* = 15), this analysis was exploratory and underpowered (<80%) to detect small-to-moderate effect sizes. Wide confidence intervals (e.g., perinatal mortality difference: 1.7–24.9%) preclude definitive clinical conclusions. VCI, velamentous cord insertion; NICU, neonatal intensive care unit.

## 4 Discussion

This study demonstrates that systematic second-trimester color Doppler ultrasound achieves excellent diagnostic performance for VCI detection. Through multivariate analysis, we identified three independent clinical risk factors associated with VCI—ART (adjusted OR = 2.87), maternal anemia (OR = 2.15), and short cervical length (OR = 2.34)—and developed a clinical association model. Placenta previa demonstrated a strong association with VCI (OR = 3.92) but likely reflects a shared spectrum of abnormal trophoblast implantation. VCI status was associated with substantially higher rates of adverse perinatal outcomes. Coexistent vasa previa was associated with higher event rates in this small sample (perinatal mortality 13.3% vs. 1.9% in isolated VCI), though the limited sample size (*n* = 15) produces imprecise estimates requiring validation in larger cohorts. These associations underscore the importance of risk factor-based surveillance, though causality cannot be inferred from this observational design.

The observed 94.2% diagnostic sensitivity exceeds prior reports ranging from 69 to 89% (12, 13), likely attributable to our standardized protocol employing low velocity scale settings (10–20 cm/s) and mandatory second-trimester screening. The significant decline in diagnostic accuracy after 28 weeks reflects compromised acoustic windows due to fetal crowding, decreased amniotic fluid, and increased maternal abdominal wall thickness (14). This temporal dependency carries critical implications for screening strategies: the 100% positive predictive value confirms that sonographic suspicion of VCI warrants immediate exclusion of vasa previa, whereas the 95.2% negative predictive value provides reliable reassurance when imaging is unequivocally normal within the optimal 18–28 week window (15).

Our multivariate analysis identified three independent predictors with distinct yet interconnected biological plausibility, converging on common pathways of disturbed embryo implantation, placental angiogenesis, and uteroplacental hemodynamics. ART conferred a 2.87-fold increased risk of VCI, likely reflecting multifaceted perturbations in early embryonic development. First, transcervical catheter placement may position the blastocyst toward the lower uterine segment, resulting in oblique rather than fundal implantation (16). Second, the *in vitro* culture environment and hormonal preparation cycles may alter trophoblast invasion phenotypes through epigenetic modifications, interfering with normal migration of the cord insertion toward the placental center (17). Additionally, the frequently observed asynchrony between endometrial and embryonic development in frozen–thawed cycles may restrict extravillous trophoblast invasion into the deep decidua (18). The synergistic interaction of these factors renders ART the strongest modifiable predictor in our model. The association between maternal anemia and VCI (OR = 2.15) requires cautious interpretation regarding directionality. While chronic iron deficiency could theoretically impair placental angiogenesis, reverse causality is equally plausible: marginal implantation or VCI-related placental insufficiency may predispose to antepartum hemorrhage and subsequent anemia. As a critical cofactor for cytochrome function and mitochondrial respiration, severe iron deficiency creates a chronic hypoxic intrauterine environment that upregulates hypoxia-inducible factor-1α and alters vascular endothelial growth factor signaling, thereby disrupting normal placental vascular branching patterns and compensatory angiogenesis (19, 20). These changes may lead to aberrant vessel distribution and secondary displacement of the cord insertion site. Regardless of temporal sequence, anemia represents a readily modifiable factor that warrants correction to optimize maternal-fetal oxygen delivery. The strong association between placenta previa and VCI (OR 3.92) likely represents a shared pathophysiological spectrum of abnormal trophoblast invasion and defective uteroplacental vascular remodeling. Both conditions originate from marginal implantation during early gestation, where restricted extravillous trophoblast migration and inadequate spiral artery remodeling result in eccentric placental development and abnormal fixation of the cord insertion (21). This shared molecular pathogenesis explains the significantly elevated concurrent risk of VCI among patients with placenta previa. Short cervical length (<25 mm) was independently associated with VCI (adjusted OR = 2.34, 95% CI: 1.21–4.53). This finding aligns with established evidence linking cervical length to placental implantation disorders: in placenta previa, short cervix predicts massive hemorrhage and adverse outcomes (22), and in placenta accreta spectrum, cervical length <30 mm independently predicts massive intraoperative bleeding (23). The mechanism—whether shared uterine architectural factors or secondary changes from placental dysfunction—remains to be determined. Regardless, cervical length assessment identifies a high-yield subgroup (15.0% vs. 4.2%) for targeted VCI screening without additional resources.

The elevated incidence of FGR in the VCI cohort (18.3% vs. 6.7%) can be attributed to hemodynamic inefficiency of the velamentous vascular architecture. Unlike centrally inserted cords with symmetric branching, VCI vessels traverse long membranous distances (mean 38.6 mm in our cohort) without Wharton jelly protection, resulting in increased flow resistance and vulnerability to compression (24). Recent hemodynamic studies confirm significantly reduced umbilical venous blood flow in VCI fetuses, directly correlating with impaired fetal growth (25). The mechanical vulnerability of exposed vessels explains the catastrophic risk amplification when VCI coexists with vasa previa: without the poroelastic buffering capacity of Wharton jelly (26), these vessels are susceptible to rupture during membrane rupture or cervical dilation, resulting in the observed 13.3% perinatal mortality in our vasa previa subgroup—sevenfold higher than isolated VCI (27).

These findings inform several actionable strategies for clinical practice. These findings suggest potential strategies for clinical practice if validated prospectively. First, systematic assessment of cord insertion site could be considered in routine second-trimester anatomy scans (18–24 weeks), particularly in ART pregnancies or those complicated by placenta previa, given the 3- to 4-fold increased risk observed in these subgroups. The risk factor model requires external validation before clinical use; if validated, it may facilitate identification of pregnancies warranting enhanced surveillance. Second, correction of maternal anemia should be prioritized following VCI diagnosis, as this represents a readily modifiable factor that may ameliorate placental insufficiency, though prospective interventional studies are needed to confirm this hypothesis. Third, all VCI cases require third-trimester transvaginal ultrasound to exclude vasa previa. In cases with confirmed vasa previa, individualized delivery planning considering gestational age and fetal status is warranted; however, our small sample (*n* = 15) cannot inform specific timing recommendations.

Compared with existing literature, our diagnostic sensitivity exceeds prior single-center studies, likely due to standardized Doppler protocols. The 3.3% perinatal mortality in our VCI cohort is lower than historical series (5–10%) (1), suggesting that systematic prenatal diagnosis and targeted management may improve outcomes, though our study design precludes causal inference. The association between ART and VCI (28.3% vs. 12.5%) is consistent with recent meta-analyses (28), but our study is the first to quantify the independent contribution of ART after adjusting for placenta previa and maternal age. Similarly, while prior studies identified anemia as a risk factor, we establish its independent predictive value in a multivariate context (29).

The strengths of this study include pathological confirmation of all VCI cases, age-matched controls minimizing confounding, comprehensive ultrasound measurements, and 1,000-bootstrap validation ensuring model robustness. This study represents one of the first attempts to systematically identify independent risk factors associated with VCI in singleton pregnancies and quantify ultrasound diagnostic performance. We emphasize that this case–control study was designed to identify risk factors associated with VCI rather than to develop a risk factor association model for screening. In a case–control design, outcome prevalence is artificially fixed at 50%, which biases the model intercept and precludes estimation of absolute risk or reliable calibration. Therefore, the reported C-index (0.713) indicates the model’s ability to discriminate between VCI cases and selected controls, not its predictive accuracy in a general obstetric population. The identified risk factors (ART, anemia, short cervix) provide valuable insights into VCI pathophysiology and may inform targeted screening strategies in high-risk subgroups. However, the coefficients from this association model cannot be directly translated into a risk scoring system for clinical use without prospective cohort validation. Future research should validate these risk factors in a prospective cohort with true VCI prevalence to develop a properly calibrated risk factor model with clinical utility. Additionally, the retrospective, single-center design introduces selection bias; our tertiary center setting may limit generalizability to community hospitals. Consequently, the reported diagnostic performance (94.2% sensitivity, 100% specificity) reflects expert-targeted assessment rather than real-world population screening, as verification bias (systematic exclusion of false negatives without prenatal suspicion), spectrum bias (enriched disease severity in referred cases), and operator expertise bias (experienced sonographers with dedicated examination time) likely inflate accuracy estimates compared to routine practice. The vasa previa subgroup analysis (*n* = 15) was underpowered (<80%) to detect small-to-moderate effect sizes. While we identified associations between anemia and short cervical length with VCI, the cross-sectional design cannot establish causality or assess whether correction of these factors improves outcomes. We lacked placental histopathological data to directly validate mechanisms involving trophoblast invasion or vascular remodeling.

## 5 Conclusion

In tertiary referral settings with expert operators, targeted color Doppler ultrasound demonstrates high diagnostic accuracy for VCI, particularly during second-trimester evaluation. The identified risk factors provide insights into VCI pathophysiology and may inform risk-based screening strategies, though prospective cohort validation is required before clinical implementation—particularly for the vasa previa subgroup analysis, which is exploratory. Integration of systematic screening with mechanism-informed risk management holds promise for reducing the substantial burden of adverse perinatal outcomes associated with this placental anomaly.

## Data Availability

The original contributions presented in the study are included in the article/Supplementary material, further inquiries can be directed to the corresponding author.
