# Supplementary material for: Velamentous cord insertion in singleton pregnancies: ultrasound diagnostic accuracy, risk factor analysis, and perinatal outcomes
Source: Front Med (Lausanne). 2026 Apr 1;13:1802669. doi: 10.3389/fmed.2026.1802669 (PMC13079321; doi:10.3389/fmed.2026.1802669)
Supplement: Supplementary file 1 [file Table_1.DOCX]

**Supplementary Table 1**.**** Comparison of Effect Estimates: Complete-Case Analysis vs. Multiple Imputation (m=5)

| Variable | Complete-case Analysis (n=240) | Multiple Imputation (n=253) | Relative Difference | P-value* |
| --- | --- | --- | --- | --- |
| **Assisted Reproductive Technology** | 2.87 (1.48–5.56) | 2.79 (1.45–5.38) | -2.8% | 0.003 |
| **Maternal Anemia (Hb <100 g/L)** | 2.15 (1.14–4.05) | 2.08 (1.12–3.87) | -3.3% | 0.021 |
| **Placenta Previa** | 3.92 (1.82–8.43) | 3.85 (1.79–8.29) | -1.8% | <0.001 |
| **Short Cervix (<25 mm)** | 2.34 (1.21–4.53) | 2.41 (1.25–4.65) | +3.0% | 0.009 |
| **Model C-index (95% CI)** | 0.713 (0.648–0.778) | 0.709 (0.645–0.773) | -0.6% | - |

*Data are adjusted odds ratios (OR, 95% CI) unless otherwise specified. P-values are from pooled analyses of 5 imputed datasets using Rubin's rules.
